# Modular metabolic engineering of *Bacillus amyloliquefaciens* for high-level production of green biosurfactant iturin A

**DOI:** 10.1007/s00253-024-13083-9

**Published:** 2024-04-27

**Authors:** Menglin She, Huijuan Zhou, Wanrong Dong, Yuxiang Xu, Lin Gao, Jiaming Gao, Yong Yang, Zhifan Yang, Dongbo Cai, Shouwen Chen

**Affiliations:** 1 State Key Laboratory of Biocatalysis and Enzyme Engineering, Environmental Microbial Technology Center of Hubei Province, College of Life Sciences, Hubei University, 368 Youyi Avenue, Wuchang District, Wuhan, 430062 Hubei People’s Republic of China; 2 Tobacco Research Institute, Chinese Academy of Agricultural Sciences, Qingdao, 266101 People’s Republic of China; 3 Hubei Corporation of China National Tobacco Corporation, Wuhan, 430000 People’s Republic of China

**Keywords:** Iturin A, *Bacillus amyloliquefaciens*, Synthetase gene cluster, Precursor supply, Metabolic engineering

## Abstract

**Abstract:**

As a kind of biosurfactants, iturin A has attracted people’s wide attentions due to their features of biodegradability, environmentally friendly, etc.; however, high production cost limited its extensive application, and the aim of this research wants to improve iturin A production in *Bacillus amyloliquefaciens*. Firstly, dual promoter was applied to strengthen iturin A synthetase expression, and its yield was increased to 1.25 g/L. Subsequently, original 5′-UTRs of downstream genes (*ituA*, *ituB*, and *ituC*) in iturin A synthetase cluster were optimized, which significantly increased mRNA secondary stability, and iturin A yield produced by resultant strain HZ-T3 reached 2.32 g/L. Secondly, synthetic pathway of α-glucosidase inhibitor 1-deoxynojirimycin was blocked to improve substrate corn starch utilization, and iturin A yield was increased by 34.91% to 3.13 g/L. Thirdly, efficient precursor (fatty acids, Ser, and Pro) supplies were proven as the critical role in iturin A synthesis, and 5.52 g/L iturin A was attained by resultant strain, through overexpressing *yngH*, *serC*, and introducing *ocD*. Meanwhile, genes responsible for poly-γ-glutamic acid, extracellular polysaccharide, and surfactin syntheses were deleted, which led to a 30.98% increase of iturin A yield. Finally, lipopeptide transporters were screened, and iturin A yield was increased by 17.98% in SwrC overexpression strain, reached 8.53 g/L, which is the highest yield of iturin A ever reported. This study laid a foundation for industrial production and application development of iturin A, and provided the guidance of metabolic engineering breeding for efficient production of other metabolites synthesized by non-ribosomal peptide synthetase.

**Key points:**

*• Optimizing 5′-UTR is an effective tactics to regulate synthetase cluster expression.*

*• Blocking 1-DNJ synthesis benefited corn starch utilization and iturin A production.*

*• The iturin A yield attained in this work was the highest yield reported so far.*

**Supplementary Information:**

The online version contains supplementary material available at 10.1007/s00253-024-13083-9.

## Introduction

Surfactant is an important component of chemical products in our daily life, and is widely used in the manufactures of detergents, washing and cleaning products, cosmetics, etc. In 2019, the market of surfactants was nearly 40 billion USD, and is expected to achieve 52 billion USD in 2025 (Gudina and Teixeira 2022). The most of surfactants commercialized nowadays are synthetic and semi-synthetic; they and their breakdown products are toxic to living organisms and cause environment pollution (Dierickx et al. 2022). Thus, eco-friendly biosurfactants produced by microorganisms attracted the widespread attentions, due to their features of biodegradability, lower toxicity, better environmentally compatibility, etc., which are considered as the potential substituted synthetic surfactants in various fields. Biosurfactants are mainly produced by *Bacillus*, and classified into three main families, surfactin (surfactin, lichenysin, pumilacidin), fengycin (fengycin, plipastation), and iturin (iturin, bacillomycin, mycosubtilin) (Ines and Dhouha 2015).

Iturin A is a kind of biosurfactant composed of hydrophobic fatty acids (14–17 carbon atoms) and hydrophilic peptide chains (L-Asn-D-Tyr-D-Asn-L-Gln-L-Pro-D-Asn-L-Ser), and the amphiphilic structure confers it strong surface and broad-spectrum activities (Tsuge et al. 2001). Now, iturin A was used as oil-displacing agent for oil recovery and potential biocontrol agent against harmful plant pathogens (Mizumoto et al. 2007; Yaraguppi et al. 2022). Four genes, *ituD*, *ituA*, *ituB* and *ituC*, are contained in iturin A synthetase cluster, and all of which are driven by promoter P_itu_. Among these genes, *ituD* encodes malonyl-CoA transacylase for fatty acid synthesis, *ituA* is responsible for β-amino fatty acid synthesis, and *ituB* and *ituC* encode peptide synthetases (Tsuge et al. 2001). It is important to note that despite the advantages of biosurfactants, high production cost served as the main factor that limited their extensive applications, which should be significantly reduced to improve their competitive (Gudina and Teixeira 2022).

The biomanufacturing revolution driven by synthetic biology is playing as the critical role in chemical, energy, agriculture, food, and other fields (Lee et al. 2005), and various approaches were also applied for biosurfactant production (Wang and Qi 2009), which were classified into the following categories: (i) strengthening synthetase cluster, (ii) enhancing precursor amino acids and fatty acids supplies, (iii) rewiring the expression of transcription factors, (iv) blocking synthetic pathways of by-products, and (v) strengthening lipopeptide exporter (Xia and Wen 2022). With the help of these strategies, the yield of surfactin was increased to 12.8 g/L, on the basis of *Bacillus subtilis* 168 that does not synthesize surfactin (Wu et al. 2019). Promoter optimization was regarded as the effective approach for metabolite synthesis; through promoter replacement and optimization, surfactin yield was increased to 9.74 g/L in *B. subtilis* (Jiao et al. 2017). As for iturin A, the promoter of iturin A synthase operon was replaced by promoter P_bacA_ in *B. amyloliquefaciens* HZ-12, which led to 1.3-fold increases of iturin A yield in the previous work of our group (Xu et al. 2020). Meanwhile, strengthening fatty acid synthesis via overexpression of acetyl-CoA carboxylase AccAD, ACP S-malonyltransferase FabD, soluble acyl ACP thioesterase TesA, and long-chain fatty acid-CoA ligase LcfA benefited iturin A synthesis, which yield was increased to 2.96 g/L by 2.95-fold (Gao et al. 2022). Although various works have been done for efficient synthesis of iturin A, its yield (2 ~ 4 g/L) was still significantly lower than that of surfactin (> 10 g/L) (Table S1), which limits iturin A application development.

**Fig. 1:**
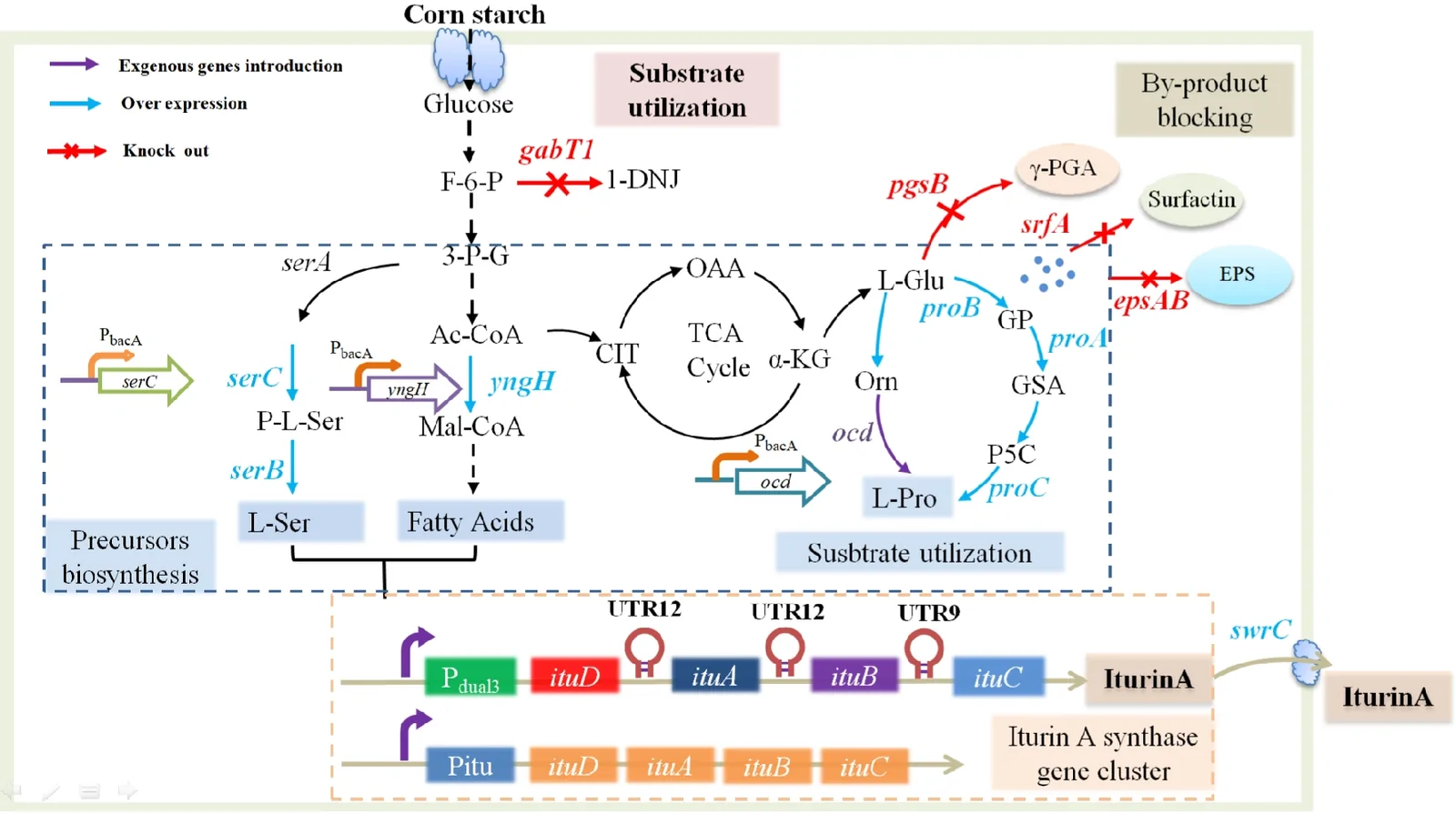
Modular metabolic engineering of *B. amyloliquefaciens* HZ-12 for enhanced production of iturin A, including the modules of iturin A synthase gene cluster, substrate utilization, precursor (fatty acids, Ser, and Pro) supplies, by-product syntheses, and iturin A transport

*B. amyloliquefaciens* HZ-12 is a non-pathogenic strain for α-glucosidase inhibitor 1-deoxynojirimycin (1-DNJ) production, and it also owns the synthesis capability of iturin A (Cai et al. 2017). In the previous research of our group, strengthening iturin A synthetase cluster and fatty acids supplies were conducted for iturin A production (Gao et al. 2022; Xu et al. 2020). In this research, to increase iturin A production, the modules of iturin A synthetase gene cluster, fatty acid and precursor amino acid supplies, substrate corn starch utilization, by-product (poly-γ-glutamic acid (γ-PGA), surfactin, extracellular polysaccharide) synthesis, and iturin A transport were engineered for strain optimization (Fig. 1). This study provided a promising strain for industrial iturin A production, and also provides the guidance of metabolic engineering breeding for efficient production of other metabolites synthesized by non-ribosomal peptide synthetase.

## Materials and methods

### Strains, medium, and cultivation conditions

The strains and plasmids used in this research were provided in Table 1. *B. amyloliquefaciens* HZ-12 (CCTCC M2015234) was applied as the original strain for recombinant strain construction; plasmid T2(2)-Ori was used for promoter replacement, gene deletion, and integration. The primers used for strain construction and RT-qPCR were listed in Table S1 (seeing in the Supplementary Materials). Lysogeny Broth (LB) medium was applied as basic medium for strain cultivation, and corresponding antibiotic (25 mg/L kanamycin or 20 mg/L tetracycline) was added, when necessary. The medium for iturin A production consisted (g/L) 30 corn starch, 70 soybean meal, 10 peanut meal, 1.0 K_2_HPO_4_·3H_2_O, 0.5 MgSO_4_·7H_2_O, 0.2 FeSO_4_·7H_2_O, 0.02 MnSO_4_·H_2_O, and natural pH. For iturin A production, the seed was cultivated in a 250-mL flask containing 30-mL LB medium for 10 h, and transferred (0.6 mL) into a 250-mL flask containing 20-mL iturin A production medium, and further cultivated at 28 °C, 230 rpm for 72 h. All the fermentation experiments were performed in triplicate.

**Table 1:** The strains and plasmids used in this research

Strains or plasmids	Relevant genotype/description	Sources
Strains		
*B. amyloliquefaciens* HZ-12	Iturin A production strain, wild-type (CCTCC M2015234)	Cai et al. (2017)
HZ-T1 (HZ-Pdual3-*ituD*)	A derivative of HZ-12, the promoter of gene *ituDABC* was replaced by P_dual3_	This work
HZ-PbacA	A derivative of HZ-12, the promoter of gene *ituDABC* was replaced by P_bacA_	Xu et al. (2020)
HZ-T2 (HZ-Pdual3-*ituD*-UTR_UTR12_-*ituA*)	A derivative of HZ-T1, the 5′-UTR of *ituA* was replaced by UTR12 in HZ-T1	This work
HZ-T3 (HZ-Pdual3-*ituD*-UTR_UTR12_-*ituA-*UTR_UTR12_-*ituB-*UTR_UTR9_-*ituC*)	A derivative of HZ-T2, the 5′-UTRs of *ituB* and ituC were, respectively, replaced by UTR12 and UTR9 in HZ-T2	This work
HZ-T4 (HZ-Pdual3-*ituD*-UTR_UTR12_-*ituA-*UTR_UTR12_-*ituB-*UTR_UTR9_-*ituC*△*gabT1*)	A derivative of HZ-T3, deletion of *gabT1* in HZ-T3	This work
HZ-T5 (HZ-Pdual3-*ituD*-UTR_UTR12_-*ituA-*UTR_UTR12_-*ituB-*UTR_UTR9_-*ituC*△*gabT1*-P43-*yngH*)	A derivative of HZ-T4, the promoter of gene *yngH* was replaced by P_43_ in HZ-T4	This work
HZ-T6 (HZ-Pdual3-*ituD*-UTR_UTR12_-*ituA-*UTR_UTR12_-*ituB-*UTR_UTR9_-*ituC*△*gabT1*-PbacA-*yngH*)	A derivative of HZ-T4, the promoter of gene *yngH* was replaced by P_bacA_ in HZ-T4	This work
HZ-T7 (HZ-Pdual3-*ituD*-UTR_UTR12_-*ituA-*UTR_UTR12_-*ituB-*UTR_UTR9_-*ituC*△*gabT1*-Pdual3-*yngH*)	A derivative of HZ-T4, the promoter of gene *yngH* was replaced by P_dual3_ in HZ-T4	This work
HZ-T8 (HZ-Pdual3-*ituD*-UTR_UTR12_-*ituA-*UTR_UTR12_-*ituB-*UTR_UTR9_-*ituC*△*gabT1*-PbacA-*yngH*-PbacA-*serC*)	A derivative of HZ-T6, the promoter of gene *serC* was replaced by P_bacA_ in HZ-T6	This work
HZ-T9 (HZ-Pdual3-*ituD*-UTR_UTR12_-*ituA-*UTR_UTR12_-*ituB-*UTR_UTR9_-*ituC*△*gabT1*-PbacA-*yngH*-PbacA-*serB*)	A derivative of HZ-T6, the promoter of gene *serB* was replaced by P_bacA_ in HZ-T6	This work
HZ-T10 (HZ-Pdual3-*ituD*-UTR_UTR12_-*ituA-*UTR_UTR12_-*ituB-*UTR_UTR9_-*ituC*△*gabT1*-PbacA-*yngH*-PbacA-*serC-PbacA-proABC*)	A derivative of HZ-T8, the promoter of gene *proABC* was replaced by P_bacA_ in HZ-T8	This work
HZ-T11 (HZ-Pdual3-*ituD*-UTR_UTR12_-*ituA-*UTR_UTR12_-*ituB-*UTR_UTR9_-*ituC*△*gabT1*-PbacA-*yngH*-PbacA-*serC-PbacA-ocd*)	A derivative of HZ-T8, introduction of gene *ocd* expression cassette in HZ-T8	This work
HZ-T12 (HZ-Pdual3-*ituD*-UTR_UTR12_-*ituA-*UTR_UTR12_-*ituB-*UTR_UTR9_-*ituC*△*gabT1*-PbacA-*yngH*-PbacA-*serC-PbacA-ocd*△*pgsB*)	A derivative of HZ-T11, deletion of gene *pgsB* in HZ-T11	This work
HZ-T13 HZ-T12 (HZ-Pdual3-*ituD*-UTR_UTR12_-*ituA-*UTR_UTR12_-*ituB-*UTR_UTR9_-*ituC*△*gabT1*-PbacA-*yngH*-PbacA-*serC-PbacA-ocd*△*pgsB*△*epsAB*△*srfA*)	A derivative of HZ-T12, deletion of genes *epsAB* and *srfA* in HZ-T12	This work
HZ-T13/pHY-300	A derivative of HZ-T13, HZ-T13 harbors plasmid pHY-300	This work
HZ-T13/pHY-YcxA	A derivative of HZ-T13, HZ-T13 harbors plasmid pHY-YcxA	This work
HZ-T13/pHY-KrsE	A derivative of HZ-T13, HZ-T13 harbors plasmid pHY-KrsE	This work
HZ-T13/pHY-SwrC	A derivative of HZ-T13, HZ-T13 harbors plasmid pHY-SwrC	This work
HZ-T14 (HZ-Pdual3-*ituD*-UTR_UTR12_-*ituA-*UTR_UTR12_-*ituB-*UTR_UTR9_-*ituC*△*gabT1*-PbacA-*yngH*-PbacA-*serC-*PbacA*-ocd*△*pgsB*△*epsAB*△*srfA-*PbacA*-swrC*)	A derivative of HZ-T13, the promoter of gene *swrC* was replaced by P_bacA_ in HZ-T13	This work
Plasmids		
T_2_(2)-Ori	*E. coli*-*Bacillus* shuttle vector, Ori_pUC_/Ori_ts_, Kan^r^	Xu et al. (2020)
T2-P_dual3_-ituD	T_2_(2)-Ori harbors promoter P_dual3_, the upstream and downstream homogenous arms of promoter P_itu_	This work
T2-UTR12-ituA	T_2_(2)-Ori harbors UTR12, the upstream and downstream homogenous arms of 5′-UTR of gene *ituA*	This work
T2-UTR12-ituB	T_2_(2)-Ori harbors UTR12, the upstream and downstream homogenous arms of 5′-UTR of gene *ituB*	This work
T2-UTR12-ituC	T_2_(2)-Ori harbors UTR9, the upstream and downstream homogenous arms of 5′-UTR of gene *ituC*	This work
T2-△gabT1	T_2_(2)-Ori harbors the upstream and downstream homogenous arms gene *gabT1*	This work
T2-P_43_-*yngH*	T_2_(2)-Ori harbors promoter P_43_, upstream and downstream homogenous arms of P_yngH_	This work
T2-P_bacA_-*yngH*	T_2_(2)-Ori harbors promoter P_bacA_, upstream and downstream homogenous arms of P_yngH_	This work
T2-P_dual3_-*yngH*	T_2_(2)-Ori harbors promoter P_dual3_, upstream and downstream homogenous arms of P_yngH_	This work
T2-P_bacA_-*serC*	T_2_(2)-Ori harbors promoter P_bacA_, upstream and downstream homogenous arms of P_serC_	This work
T2-P_bacA_-*serB*	T_2_(2)-Ori harbors promoter P_bacA_, upstream and downstream homogenous arms of P_serB_	This work
T2-P_bacA_-*proABC*	T_2_(2)-Ori harbors promoter P_bacA_, upstream and downstream homogenous arms of P_proABC_	This work
T2-ocd	T_2_(2)-Ori harbors promoter P_bacA_, gene *ocd* from *Bacillus thuringiensis*, and *amyL* terminator	This work
T2-*pgsB*	T_2_(2)-Ori upstream and downstream homogenous arms of *pgsB*	This work
T2-*srfA*	T_2_(2)-Ori upstream and downstream homogenous arms of *srfA*	This work
T2-*eps*	T_2_(2)-Ori upstream and downstream homogenous arms of *eps*	This work
pHY300PLK	*E. coli* and *B. subtilis* shuttle vector; Amp^r^, Tet^r^	Xu et al. (2020)
pHY-YcxA	Gene *ycxA* expression vector, based on pHY300PLK	This study
pHY-KrsE	Gene *krsE* expression vector, based on pHY300PLK	This study
pHY-SwrC	Gene *swrC* expression vector, based on pHY300PLK	This study
T2-P_bacA_-SwrC	T_2_(2)-Ori harbors promoter P_bacA_, upstream and downstream homogenous arms of P_swrC_	This study

### Recombinant strain construction in *B. amyloliquefaciens*

The promoter replacement, gene deletion, and integration in *B. amyloliquefaciens* HZ-12 were carried out using temperature-sensitive shuttle plasmid T2(2)-Ori (Cai et al. 2018). As for promoter replacement, the construction procedure of promoter P_itu_ replacement strain was served as an example. Briefly, corresponding primers were applied to amplify promoter P_dual3_, upstream and downstream homologous arms of promoter P_itu_, and fused by splicing overlap extension (SOE)-PCR. The fusion fragment was digested and inserted into *Xba*I/*Sac*I sites of T2(2)-Ori. Diagnostic PCR and DNA sequence confirmed the successful construction of promoter replacement plasmid, named as T2-P_dual3_-ituD. Then, T2-P_dual3_-ituD was transferred into *B. amyloliquefaciens* by electroporation, and promoter replacement strain was attained by homologous double exchanges; diagnostic PCR and DNA sequence confirmed that the promoter replacement strain was successfully constructed. Similarly, construction procedures of gene deletion and integration strains were referred to that of promoter replacement strain, verified by diagnostic PCR and DNA sequence.

Plasmid pHY300PLK was applied for gene overexpression strain construction, and construction procedure of gene *ycxA* overexpression strain was served as an example (Gao et al. 2022). Briefly, promoter P_bacA_ (Genebank ID 16053260), gene *ycxA* (14,767,903), and *amyL* terminator (16,055,532) were amplified and fused by SOE-PCR; the fusion fragment was inserted into pHY300PLK at *Eco*RI/*Sac*I sites; and diagnostic PCR and DNA sequence confirmed that YcxA overexpression vector pHY-YcxA was successfully constructed. Then, pHY-YcxA was electro-transferred into *B. amyloliquefaciens* to construct YcxA overexpression strain, and other gene overexpression strains were attained by the same method.

### Analytical methods

Cell biomass was measured by dilution plating. To determine the concentration of iturin A, the volume of 0.3-mL fermentation supernatant was mixed with 1.2 mL methanol, shaken for 1 h, and centrifuged at 10,000 *g* for 10 min, and the supernatant was attained for iturin A determination. The iturin A concentration was measured on an Agilent HPLC 1260 (Agilent Technologies, USA), equipped with Agilent Lichrospher C18 column (4.6 mm × 250 mm, 5 μm). The mobile phase was 10 mM ammonium acetate/acetonitrile = 65:35 (V/V), and flow rate was 1.0 mL/min. The injection volume was 10 μL, and detection wavelength was 210 nm. Iturin A concentration was calculated by the curve made by iturin A standard (Sigma, CAS 52229–90-0) (Gao et al. 2022). In addition, mRNA secondary structures of 5′-UTRs were predicted by software Mfold (http://unafold.rna.albany.edu/?q=mfold/RNA-Folding-Form).

The concentrations of 1-DNJ, γ-PGA, extracellular polysaccharide, and surfactin were measured by HPLC, according to the previously reported methods (Cai et al. 2018, 2017; Wu et al. 2019). Gas chromatography was applied to determine the contents of amino acids, according to the previous research (Zhu et al. 2021). Fatty acids were extracted and derived by n-hexane, and the contents of fatty acids were measured by Gas Chromatography Mass Spectrometry (GC–MS), equipped with TG-5MS column (30 m × 0.25 mm, Thermo), and the contents of free fatty acids were determined by previously reported method (Gao et al. 2022).

### Transcriptional-level analysis

The transcriptional levels of genes were determined according to the previous research (Zhan et al. 2022). RNA was extracted by TRIzol® Reagent, and the first stand of cDNA was amplified by Revert Aid First-Strand cDNA Synthesis Kit (Thermo, USA). Gene transcriptional levels of recombinant strains were compared with those of control strain, after being normalized to reference gene *16S rRNA*.

### Statistic analysis

All experiments were repeated at least three times, and data were presented as the mean ± standard deviation for each sample point. All data were conducted to analyze the variance at *p* < 0.05 (*) and *p* < 0.01 (**), and mean values were compared by applying a *t* test, using the software package Statistica 6.0 (Cai et al. 2018).

## Results

### Manipulating iturin A synthetase gene cluster for iturin A production

**Fig. 2:**
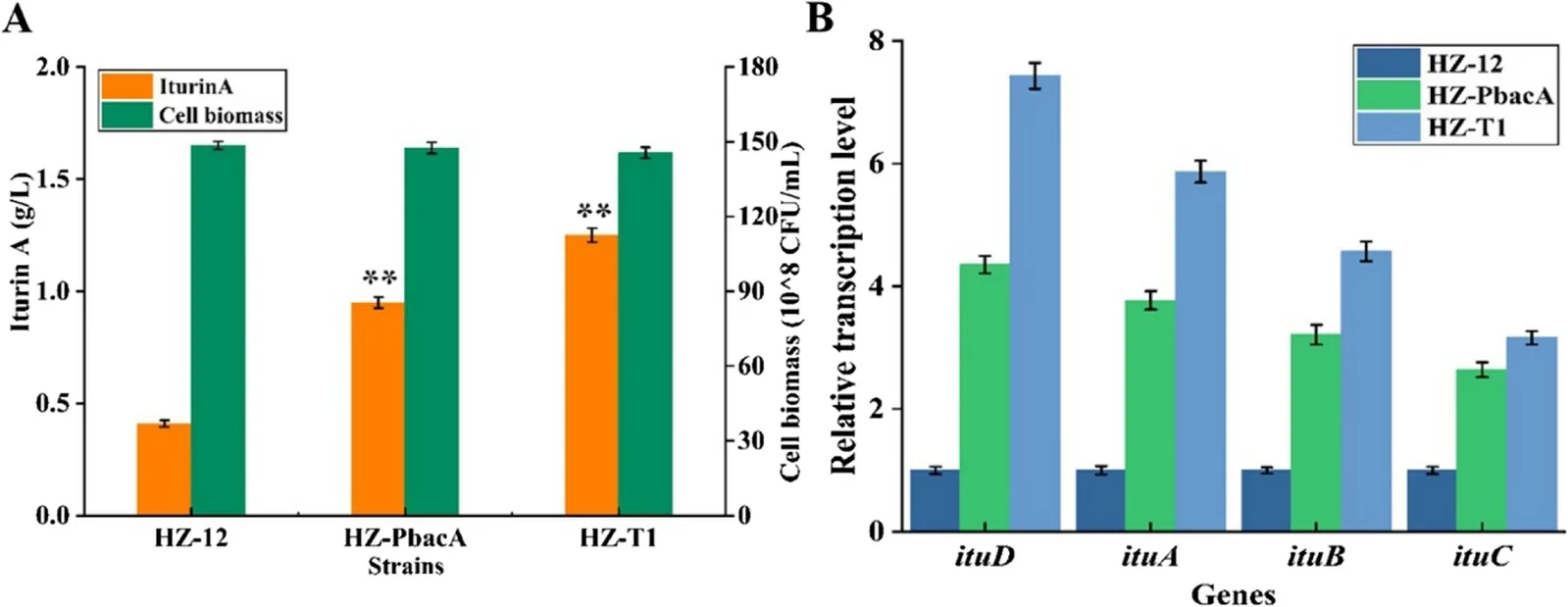
Strengthening iturin A synthetase cluster expression in HZ-12 (wild-type strain) for iturin A production. **A** Iturin A yield and cell biomass and **B** transcriptional levels of genes *ituA*, *ituB*, *ituC*, and *ituD*

Promoter replacement is an effective strategy for gene expression regulation, which has been applied in the efficient production of various lipopeptides (surfactin, lichenysin, fenycin, and iturin A) (Jiao et al. 2017; Qiu et al. 2014; Xu et al. 2020; Yaseen et al. 2016). The dual promoter P_dual3_ constructed in our previous research has been proven as an effective promoter in gene expression enhancement (Rao et al. 2020). Here, the original promoter (P_itu_) of iturin A synthase gene cluster was replaced by P_dual3_, obtained recombinant strain HZ-T1 (HZ-Pdual3-*ituD*), and strain HZ-PbacA, in which P_itu_ was replaced by promoter P_bacA_ in our previous research (Xu et al. 2020), was served as the control strain, as well as original strain HZ-12. Based on our results of Fig. 2, iturin A yields were significantly increased in promoter replacement strains, and the highest yield was attained by HZ-T1, reached 1.25 g/L, increased by 2.05-fold compared to HZ-12 (Fig. 2A). In addition, transcription levels of genes *ituD*, *ituA*, *ituB*, and *ituC* were all increased at logarithmic and stationary phases (Fig. 2B), and increase ratios of downstream genes (*ituA*, *ituB*, and *ituC*) were lower than those of *ituD*.

**Fig. 3:**
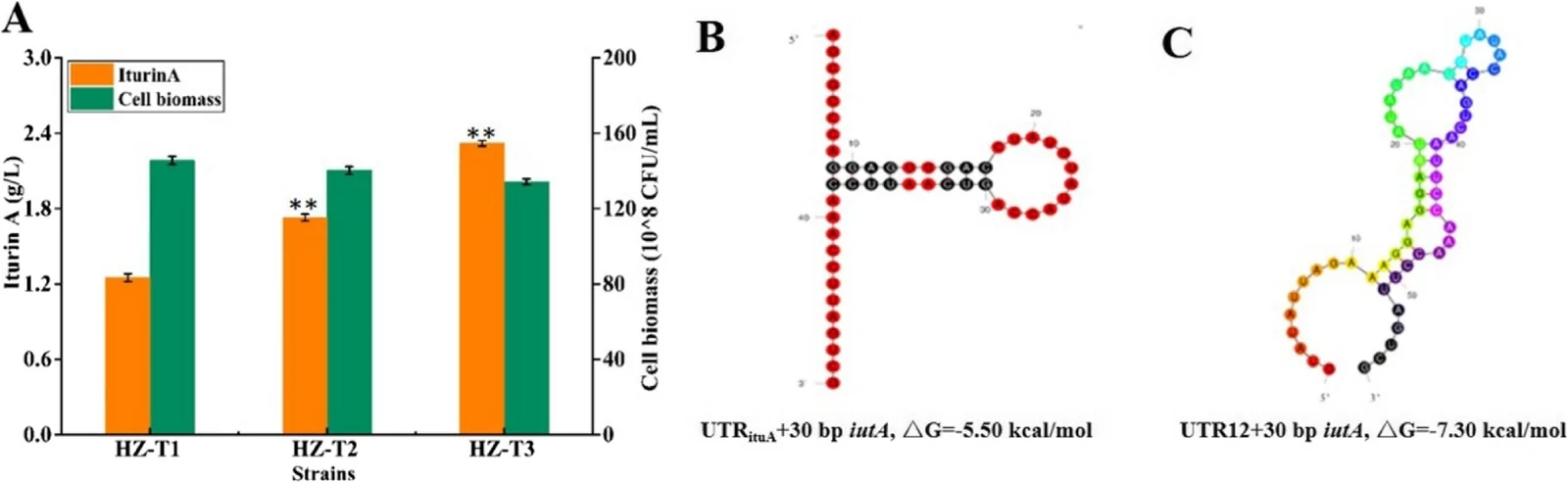
Optimizing the 5′-UTRs of genes *ituB*, *ituC*, and *ituD* in HZ-T1 (HZ-Pdual3-*ituD*) for enhanced production of iturin A. **A** Iturin A yield and cell biomass, **B** mRNA secondary structure of gene *ituB* with its original 5′-UTR, and **C** the mRNA secondary structure of UTR12 with gene *ituB*

5′-UTR served as the critical role in mRNA secondary structure stability and translation initiation (Xiao et al. 2020). Here, to further improve genes *ituA*, *ituB*, and *ituC* expression for iturin A synthesis, the original 5′-UTRs of these genes were optimized in the following work. Firstly, 5′-UTR of gene *ituA* in HZ-T1 was replaced by UTR12 (GTATATTAGAAAGGAGGAATATATA), which was attained in our previous research (Xiao et al. 2020), and iturin A yield produced by the resultant strain HZ-T2 was 1.73 g/L, increased by 38.40% compared to HZ-T1 (Fig. 3A). Meanwhile, the mRNA secondary structures of 5′-UTRs with the first 30 bp of gene *ituA* were predicted on Mfold program, and our results implied that 5′-UTR replacement decreased Δ*G* of mRNA secondary structure, which benefited mRNA secondary structure stability and gene expression (Fig. 3 B and C), and this might be reason for the increase of iturin A yield in HZ-T2 (HZ-Pdual3-*ituD*-UTR_UTR12_-*ituA*). Then, original 5′-UTRs of *ituB* and *ituC* were, respectively, replaced by UTR12 and UTR9 (GGTACATTAGAAAGGAGGAATGTACC) in HZ-T2, basing on the matching between 5′-UTR with relative gene sequences (Figure S1), and iturin A yield of the recombinant strain HZ-T3 (HZ-Pdual3-*ituD*-UTR_UTR12_-*ituA-*UTR_UTR12_-*ituB-*UTR_UTR9_-*ituC*) was further increased to 2.32 g/L, increased by 85.60% compared to HZ-T1 (Fig. 3A). Thus, our results implied that 5′-UTR optimization was an efficient approach to regulate the expression of downstream genes in synthetase cluster.

### Enhancing the substrate utilization for iturin A synthesis

**Fig. 4:**
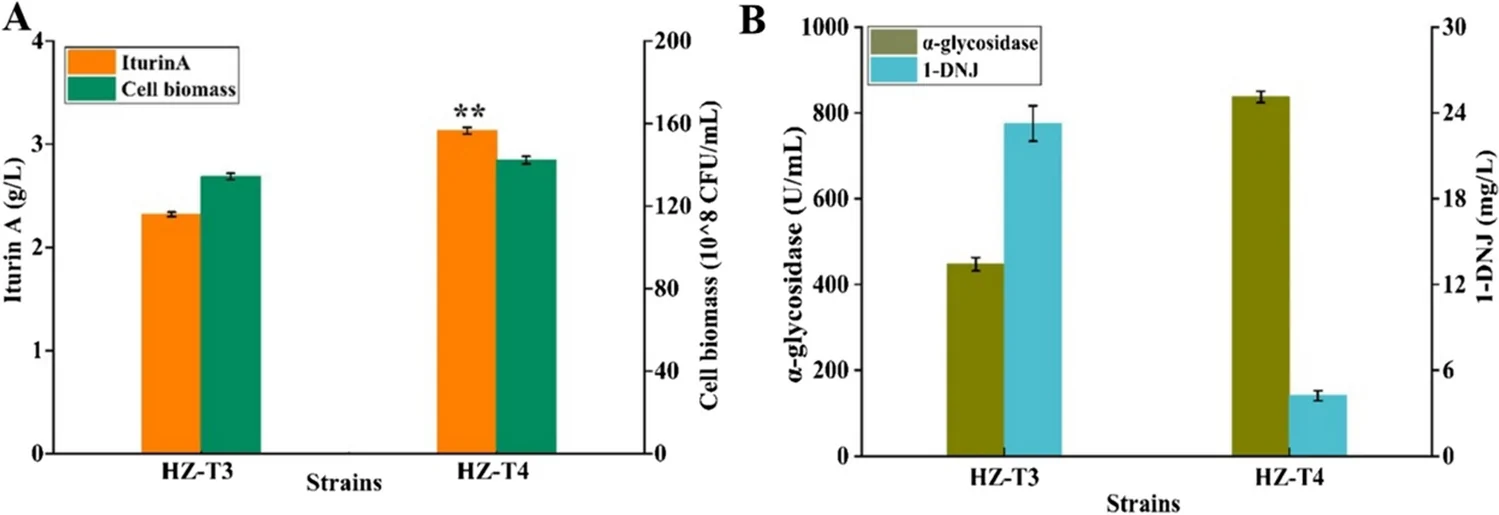
Improving corn starch utilization by blocking 1-DNJ synthesis pathway in HZ-T3 (HZ-Pdual3-*ituD*-UTR_UTR12_-*ituA-*UTR_UTR12_-*ituB-*UTR_UTR9_-*ituC*) to increase iturin A production. **A** Iturin A yield and cell biomass and **B** 1-DNJ yield and α-glycosidase activity

In the previous research of our group, *B. amyloliquefaciens* HZ-12 was attained for the production of α-glucosidase inhibitor 1-DNJ (Cai et al. 2017); however, 1-DNJ produced by HZ-T3 might decrease α-glucosidase activity, which was not conducive to corn starch utilization and iturin A synthesis. 4-Aminobutyrate transaminase encoded by gene *gabT1* catalyzes the critical step of 1-DNJ synthesis (Onose et al. 2013); here, *gabT1* was deleted in strain HZ-T3 to attain HZ-T4 (HZ-Pdual3-*ituD*-UTR_UTR12_-*ituA-*UTR_UTR12_-*ituB-*UTR_UTR9_-*ituC*△*gabT1*). Based on the results of Fig. 4, iturin A yield produced by HZ-T4 was 3.13 g/L, increased by 34.91% and 6.63-fold compared to HZ-T3 and HZ-12, respectively (Fig. 4A). Meanwhile, 1-DNJ content of HZ-T4 was significantly decreased from 23.26 to 4.23 mg/L, while α-glucosidase activity was increased by 87.08%, respectively (Fig. 4B). In addition, the maximum cell biomass of strain HZ-T4 was higher than that of HZ-T3. Taken together, our results implied that enhancing α-glucosidase activity via blocking 1-DNJ synthetic pathway benefited corn starch utilization, which further benefited iturin A production.

### Strengthening fatty acid and precursor amino acid supplies for iturin A synthesis

**Fig. 5:**
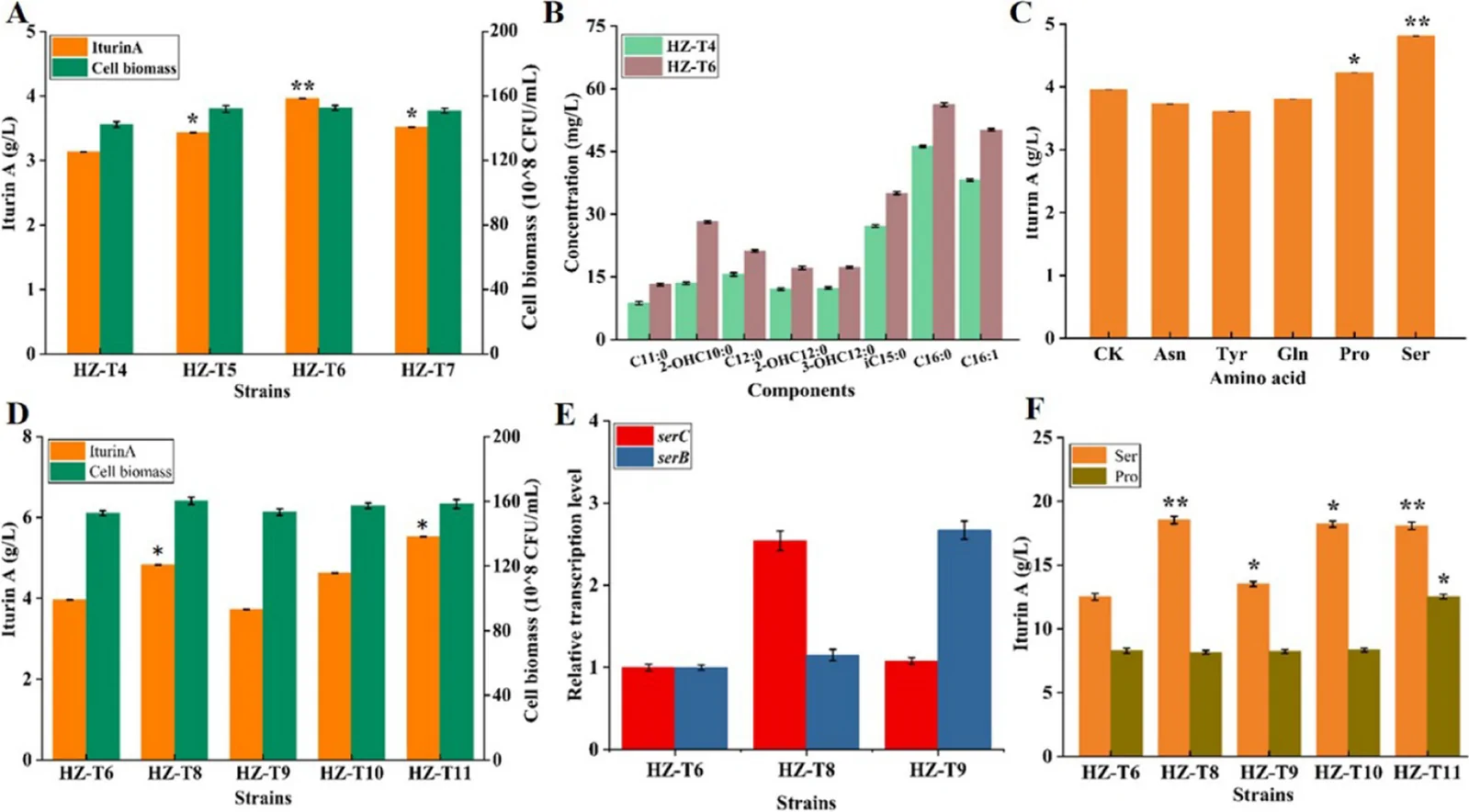
Strengthening precursor (fatty acids and amino acids) supplies in HZ-T4 (HZ-Pdual3-*ituD*-UTR_UTR12_-*ituA-*UTR_UTR12_-*ituB-*UTR_UTR9_-*ituC*△*gabT1*) for iturin A production. **A** Iturin A yield and cell biomass, **B** the concentrations of fatty acids, **C** effects of precursor amino acid additions on iturin A yield, **D** strengthening precursor Ser and Pro synthesis pathways for iturin production, **E** transcriptional levels of genes *serC* and *serB*, and **F** the concentrations of intracellular Ser and Pro

Enhancing precursor supply is a common and effective strategy for target product production (Zhu et al. 2021). As for iturin A, the accumulations of free fatty acids and precursor amino acids might be the limiting factor for its synthesis. Here, to increase fatty acid supplies, three promoters (P43, P_bacA_, and P_dual3_) were applied for *yngH* expression in HZ-T4, resulting in strains HZ-T5 (HZ-Pdual3-*ituD*-UTR_UTR12_-*ituA-*UTR_UTR12_-*ituB-*UTR_UTR9_-*ituC*△*gabT1*-P43-*yngH*), HZ-T6 (HZ-Pdual3-*ituD*-UTR_UTR12_-*ituA-*UTR_UTR12_-*ituB-*UTR_UTR9_-*ituC*△*gabT1*-PbacA-*yngH*), and HZ-T7 (HZ-Pdual3-*ituD*-UTR_UTR12_-*ituA-*UTR_UTR12_-*ituB-*UTR_UTR9_-*ituC*△*gabT1*-Pdual3-*yngH*), respectively. Basing on the results of Fig. 5, transcriptional levels of *yngH* were all increased in the promoter replacement strains (Figure S2), and iturin A yields were increased by 9.58%, 26.52%, and 12.46%, respectively, and the best performance was attained by strain HZ-T6 (Fig. 5A). In addition, the contents of free fatty acids were all increased in HZ-T6 (Fig. 5B).

Compared to surfactin, amino acid composition of iturin A was complicated, which might be the reason for low synthetic capability of iturin A in *Bacillus*. In order to excavate the limiting precursor amino acid that is hindering iturin A synthesis, 40 mg/L Asn, Tyr, Gln, Pro, and Ser were, respectively, added into iturin A production medium at 24 h, and iturin A yields were, respectively, increased by 21.46% and 6.82% in Ser- and Pro-feeding groups (Fig. 5C), suggesting that Ser and Pro might be the limiting amino acids for iturin A synthesis in HZ-T6; however, Asn, Tyr, and Gln additions have no effect on iturin A production. Then, to improve intracellular Ser supply for iturin A production, original promoters of 3-phosphoserine aminotransferase SerC and phosphoserine phosphatase SerB were, respectively, replaced by promoter P_bacA_ in HZ-T6, resulting in recombinant strains HZ-T8 (HZ-Pdual3-*ituD*-UTR_UTR12_-*ituA-*UTR_UTR12_-*ituB-*UTR_UTR9_-*ituC*△*gabT1*-PbacA-*yngH*-PbacA-*serC*) and HZ-T9 (HZ-Pdual3-*ituD*-UTR_UTR12_-*ituA-*UTR_UTR12_-*ituB-*UTR_UTR9_-*ituC*△*gabT1*-PbacA-*yngH*-PbacA-*serB*), respectively. The transcriptional level of *serC* was increased by 1.54-fold, and intracellular concentration of Ser was increased by 48.08%, which led to a 21.97% enhancement of iturin A yield. Overexpression of SerB have no effect on iturin A production, although transcription level of *serB* was enhanced in HZ-T9 (Fig. 5 D and E). In *B. amyloliquefaciens* HZ-12, Pro was synthesized from Gln, under the catalysis of gene cluster *proABC*; however, strengthening ProABC expression (HZ-T10 (HZ-Pdual3-*ituD*-UTR_UTR12_-*ituA-*UTR_UTR12_-*ituB-*UTR_UTR9_-*ituC*△*gabT1*-PbacA-*yngH*-PbacA-*serC-*PbacA-*proABC*)) has no effect on the intracellular Pro concentration and iturin A yield in this research. In addition, Pro can also be converted from ornithine, under the catalysis of ornithine cyclodeaminase Ocd in *Bacillus thuringiensis*. Here, gene *ocD* from *B. thuringiensis* BMB171 (NC_014171.1) was introduced into HZ-T8, resulting in strain HZ-T11 (HZ-Pdual3-*ituD*-UTR_UTR12_-*ituA-*UTR_UTR12_-*ituB-*UTR_UTR9_-*ituC*△*gabT1*-PbacA-*yngH*-PbacA-*serC*-PbacA-*ocd*). The concentration of intracellular Pro was increased by 53.37% in HZ-T11, and iturin A yield was increased to 5.52 g/L by 14.29% compared to HZ-T8 (Fig. 5 D and F), which was positively correlated with the previous research (Chen et al. 2022). Thus, our results demonstrated that strengthening precursor (fatty acids, Ser, and Pro) supplies was an efficient approach for iturin A production.

### Blocking the by-product synthetic pathways for iturin A synthesis

**Fig. 6:**
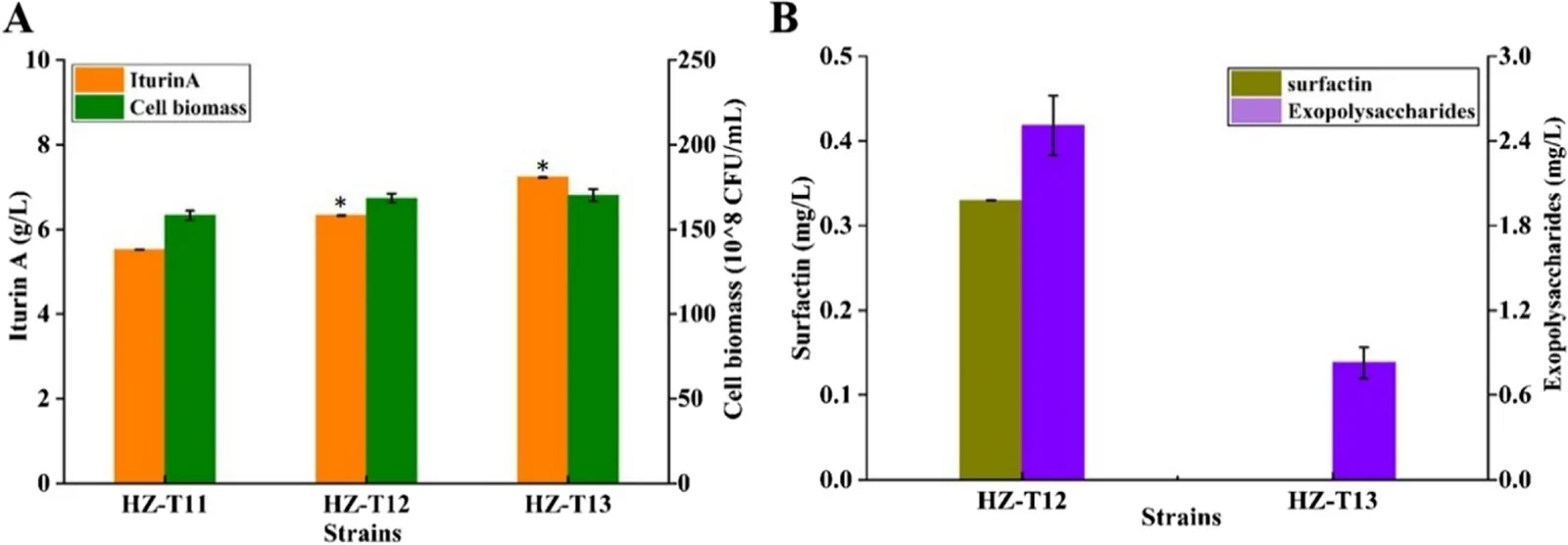
Blocking the by-product synthetic pathways in HZ-T11 (HZ-Pdual3-*ituD*-UTR_UTR12_-*ituA-*UTR_UTR12_-*ituB-*UTR_UTR9_-*ituC*△*gabT1*-PbacA-*yngH*-PbacA-*serC-*PbacA-*ocd*) for iturin A synthesis. **A** Iturin A yields and cell biomasses and **B** the yields of by-products γ-PGA, extracellular polysaccharide, and surfactin

The syntheses of by-products will not only affect the conversion ratio of raw materials, but also influence the fermentation quality, and increase the difficulty of separation and extraction, which is not conducive to the efficient production of target production. γ-PGA is a natural multi-functional biopolymer that was mainly produced by *Bacillus*, and high viscosity of γ-PGA will affect oxygen supply and product separation (Cai et al. 2018). Here, gene *pgsB* encoding for γ-PGA synthetase was deleted in HZ-T11, and iturin A produced by the resultant strain HZ-T12 (HZ-Pdual3-*ituD*-UTR_UTR12_-*ituA-*UTR_UTR12_-*ituB-*UTR_UTR9_-*ituC*△*gabT1*-PbacA-*yngH*-PbacA-*serC-*PbacA-*ocd*△*pgsB*) was 6.33 g/L, increased by 14.67% compared to HZ-T11 (Fig. 6A). Furthermore, genes *epsAB* and *srfA*, which are responsible for extracellular polysaccharide and surfactin syntheses (Wu et al. 2019), were deleted in HZ-T12 (HZ-Pdual3-*ituD*-UTR_UTR12_-*ituA-*UTR_UTR12_-*ituB-*UTR_UTR9_-*ituC*△*gabT1*-PbacA-*yngH*-PbacA-*serC-*PbacA-*ocd*△*pgsB*△*epsAB*△*srfA*), which led to the significant decreases of extracellular polysaccharide and surfactin yields (Fig. 6B), and iturin A yield was further increased by 14.22% to 7.23 g/L, compared to HZ-T12.

### Strengthening lipopeptide transporter for iturin A production

**Fig. 7:**
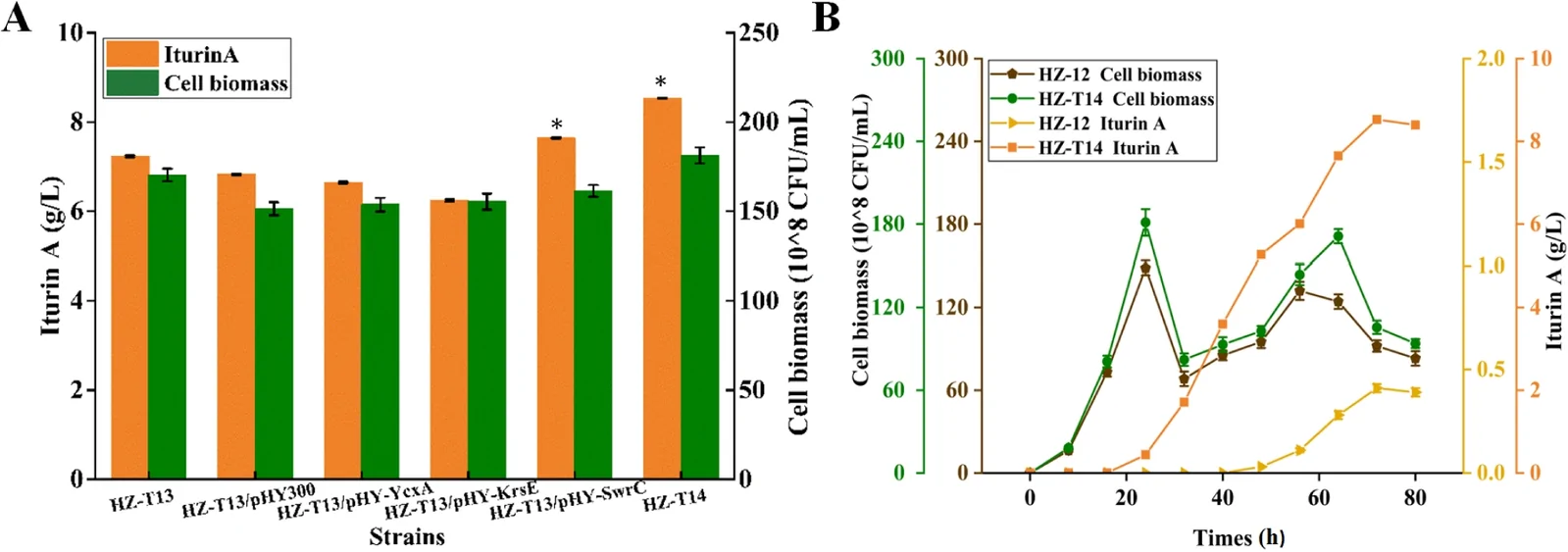
Overexpression of lipopeptide transporters in HZ-T13 (HZ-Pdual3-*ituD*-UTR_UTR12_-*ituA-*UTR_UTR12_-*ituB-*UTR_UTR9_-*ituC*△*gabT1*-PbacA-*yngH*-PbacA-*serC-PbacA-ocd*△*pgsB*△*epsAB*△*srfA*) for iturin A production. **A** Iturin A yields and cell biomasses and **B** the fermentation process curves of strains HZ-12 and HZ-T14

An excellent transporter is very important for efficient production of target products, especially for the product which inhibits cell growth and metabolism (Li et al. 2010). Previously, YcxA, KrsE, and SwrC were reported as lipopeptide exporters for surfactin production (Li et al. 2015); however, which one mediates iturin A transport is unclear. Here, YcxA, KrsE, and SwrC overexpression strains were constructed basing on HZ-T13, attained in recombinant strains HZ-T13/pHY-YcxA, HZ-T13/pHY-KrsE, and HZ-T13/pHY-SwrC. Based on the results of Fig. 7A, plasmid introduction increased cell maintain metabolite energy, which decreased iturin A production. SwrC overexpression benefited iturin A transport, and iturin A yield was increased to 7.64 g/L by 12.02%, and cell biomass was increased by 6.60%, compared to control strain HZ-T13/pHY300, respectively. However, overexpression of YcxA or KsrE has no effect on iturin A production. Subsequently, the original promoter of SwrC was replaced by promoter P_bacA_ in HZ-T13, resulting in strain HZ-T14 (HZ-Pdual3-*ituD*-UTR_UTR12_-*ituA-*UTR_UTR12_-*ituB-*UTR_UTR9_-*ituC*△*gabT1*-PbacA-*yngH*-PbacA-*serC*-PbacA-*ocd*△*pgsB*△*epsAB*△*srfA*-PbacA-*swrC*), and iturin A yield produced by HZ-T14 reached 8.53 g/L, increased by 17.98% compared to HZ-T13.

Finally, the fermentation process curves of strains HZ-12 and HZ-T14 were measured during iturin A production. Based on the results of Fig. 7B, cell biomasses of HZ-T14 were higher than those of HZ-12 throughout the fermentation process, and the maximum cell biomass was increased by 21.99%. Iturin A was synthesized after 24 h, and maximum yield of HZ-T14 reached 8.53 g/L, increased by 19.80-fold compared to HZ-12, which was the highest iturin A yield reported so far (Table S1).

## Discussions

Acting as one kind of important biosurfactants, iturin A has the wide application values in the fields of petroleum recovery and fungal disease biocontrol (Mizumoto et al. 2007; Yaraguppi et al. 2022); however, low synthetic level limits its application and popularization. *Bacillus* was proven as the efficient host for biosurfactant production (Zhao et al. 2017); here, to increase iturin A production, the modules of iturin A synthetase gene cluster, substrate corn starch utilization, precursor and by-product syntheses, and iturin A transport were manipulated, basing on *B. amyloliquefaciens* HZ-12, and the final strain HZ-T14 was constructed with iturin A yield of 8.53 g/L, which was the highest iturin A yield reported so far. Our results demonstrated that modular metabolic engineering was an effective approach for enhanced production of iturin A, and *B. amyloliquefaciens* HZ-T14 was attained with the promising application for industrial production of iturin A.

The third biotechnology revolution triggered by synthetic biology has made the great achievements in the fields of biochemistry, energy, agriculture, etc. (Lee et al. 2005; Wang and Qi 2009). Surfactants are important components in the production of numerous products in our daily life, and biosurfactants, mainly containing surfactin, lichenysin, and iturin, have attracted various attentions in recent years, as their wonderful characters of biodegrading, environmentally friendly, etc. (Gudina and Teixeira 2022; Xia and Wen 2022). Previously, various metabolic engineering approaches have been developed for surfactin production, and the yield of which was increased to more than 10 g/L (Wang et al. 2019; Wu et al. 2019). Acting as a homolog of surfactin, lichenysin yield was increased by 16.8-fold by promoter replacement and fermentation process optimization in the previous researches of our group (Hu et al. 2022; Qiu et al. 2014). Acting as the main raw materials for nylon-66 production, adipic acid served as the critical role in biochemical industry. In recent years, metabolic engineering approaches have been conducted to improve adipic acid production (Zhao et al. 2018); also, an artificial biosynthesis system was established to catalyze the synthesis reaction of cyclohexane to adipic acid in *Escherichia coli* (Wang et al. 2020). With the increasing attention to environmental protection, degradable bio-based materials are widely attracting people’s attentions (Han et al. 2022), just as the global production capacity of poly-lactic acid (PLA) is expanding. Due to their biocompatibility and biodegradability, polyhydroxyalkanoates (PHA) have the widespread applications in the areas of chemical industry, medical, etc. Through systematically modified *Halomonas*, Chen’s group has achieved the great achievements in the field of PHA synthetic biomanufacture (Zhang et al. 2020). γ-PGA can be also applied in the development of environmentally friendly flocculants, as its properties of biodegradable and heavy metal chelating. Focusing on systematic metabolic engineering and fermentation optimization of γ-PGA synthesis for nearly 20 years, our group has realized the efficient production and industrialization of γ-PGA in *Bacillus* (Zhang et al. 2022). In this research, iturin A yield was significantly increased by 19.80-fold via systematically modify the modules of iturin A synthetase cluster, substrate utilization, precursor supplies and by-product blocking, and lipopeptide transport, which laid the foundation for iturin A large-scale production and application development. Thus, the continuous innovations of synthetic biology will drive the transformative developments of biochemistry industry.

As for genome simplify and gene coordination expression, most secondary metabolite synthetase genes are presented as gene clusters, driven by a single promoter. However, this may not be appropriate to downstream gene expression, as the transcriptional levels of downstream genes were generally lower than those of upstream, which was also confirmed by our results (Fig. 2B). Therefore, the expression levels of downstream genes must be manipulated to improve gene cluster expression and target product production. Promoter replacement has been proven as an efficient approach for gene expression regulation (Rao et al. 2020); however, promoter introduction of downstream genes might cause gene expression disorder, which is not conducive to metabolite synthesis. 5′-UTR plays an important role in mRNA secondary structure stability and translation initiation, which further affects gene expression level (Xiao et al. 2020). Previously, a 5′-UTR library was constructed in our previous research (Rao et al. 2021), and it has been applied in the construction of gradient promoters for heterologous protein and metabolite syntheses. Here, in order to increase the expression levels of downstream genes for iturin A production, the original 5′-UTRs of genes *ituA*, *ituB*, and *ituC* were, respectively, replaced by UTR12, UTR12, and UTR9, basing on the matching of relative genes with 5′-UTR (Bentele et al. 2013; Rao et al. 2021; Xiao et al. 2020), and iturin A yield was increased by 85.60%. Therefore, our results demonstrated that 5′-UTR optimization is an effective approach to regulate the expression of downstream genes for metabolite production.

As a common carbon source in industrial fermentation, corn starch is applied for the production of antibiotics, enzyme proteins, etc. (Zhao et al. 2022). Compared to glucose, high concentrations of corn starch do not cause carbon catabolite repression (CCR), which avoids the problem of complex fermentation process control of glucose-feeding. Previously, although people have achieved the efficient synthesis of a variety of metabolites through systematic metabolic engineering, few strategies have been developed for efficient utilization of corn starch, as the corn starch hydrolysate was complex to be detected. To increase avermectin production, Li et al. have improved the expression level of *malEFG* by gene integration in *Streptomyces avermitilis* ATCC31267, which led to 2.6-fold increases of avermectin yield (Li et al. 2010). In this research, α-glucosidase activity was significantly increased in *gabT1* deletion strain (Onose et al. 2013), which led to a 34.91% increase of iturin A yield. In addition, rewiring the utilization modules of corn starch and its hydrolytic maltodextrin might be also an effective strategy to increase target product production, and these works are conducting for other metabolite production in our group.

In conclusion, biosurfactant iturin A has the wide applications in the fields of petrochemical, agriculture, etc., as its properties of biodegradable, environmentally friendly, antifungal, and low synthetic level hinder its application expansion. Here, to increase the synthetic capability of iturin A in *B. amyloliquefaciens*, the modules of iturin A synthetase gene cluster, corn starch utilization, precursor supplies and by-product blocking, and iturin A transport were engineered, and iturin A yield was increased by 19.80-fold to 8.53 g/L in the final strain HZ-T14, which was the highest yield of iturin A reported to date. Taken together, our results demonstrated that metabolic engineering is an effective approach for enhanced production of iturin A, and provided the guidance of metabolic engineering breeding for efficient production of other metabolites synthesized by non-ribosomal peptide synthetase.

## Supplementary information

Below is the link to the electronic supplementary material.

Supplementary file1 (PDF 343 KB)

## Data Availability

The datasets used and/or analyzed during the current study are available from the corresponding author on reasonable request.

## References

[CR1] Bentele K, Saffert P, Rauscher R, Ignatova Z, Bluthgen N (2013) Efficient translation initiation dictates codon usage at gene start. Mol Syst Biol 9:675 23774758 10.1038/msb.2013.32 PMC3964316

[CR2] Cai D, Liu M, Wei X, Li X, Wang Q, Nomura CT, Chen S (2017) Use of *Bacillus amyloliquefaciens* HZ-12 for high-level production of the blood glucose lowering compound, 1-deoxynojirimycin (DNJ), and nutraceutical enriched soybeans via fermentation. Appl Biochem Biotechnol 181(3):1108–1122 27826807 10.1007/s12010-016-2272-8

[CR3] Cai D, Chen Y, He P, Wang S, Mo F, Li X, Wang Q, Nomura CT, Wen Z, Ma X et al (2018) Enhanced production of poly-gamma-glutamic acid by improving ATP supply in metabolically engineered *Bacillus licheniformis*. Biotechnol Bioeng 115(10):2541–2553 29940069 10.1002/bit.26774

[CR4] Chen X, Sun H, Qiao B, Miao C, Hou Z, Xu S, Xu Q, Cheng J (2022) Improved the lipopeptide production of *Bacillus amyloliquefaciens* HM618 under co-culture with the recombinant *Corynebacterium glutamicum* producing high-level proline. Bioresour Technol 349:126863 35183721 10.1016/j.biortech.2022.126863

[CR5] Dierickx S, Castelein M, Remmery J, De Clercq V, Lodens S, Baccile N, De Maeseneire S, Roelants S, Soetaert W (2022) From bumblebee to bioeconomy: recent developments and perspectives for sophorolipid biosynthesis. Biotechnol Adv 54:107788 34166752 10.1016/j.biotechadv.2021.107788

[CR6] Gao L, She M, Shi J, Cai D, Wang D, Xiong M, Shen G, Gao J, Zhang M, Yang Z et al (2022) Enhanced production of iturin A by strengthening fatty acid synthesis modules in *Bacillus amyloliquefaciens*. Front Bioeng Biotechnol 10:974460 36159706 10.3389/fbioe.2022.974460 PMC9500472

[CR7] Gudina E, Teixeira J (2022) *Bacillus licheniformis*: the unexplored alternative for the anaerobic production of lipopeptide biosurfactants? Biotechnol Adv 60:108013 10.1016/j.biotechadv.2022.108013 35752271

[CR8] Han X, Liu J, Tian S, Tao F, Xu P (2022) Microbial cell factories for bio-based biodegradable plastics production. iScience 25(11):105462 36405773 10.1016/j.isci.2022.105462 PMC9667314

[CR9] Hu S, He P, Zhang Y, Jiang M, Wang Q, Yang S, Chen S (2022) Transcription factor DegU-mediated multi-pathway regulation on lichenysin biosynthesis in *Bacillus licheniformis*. Metab Eng 74:108–120 36257594 10.1016/j.ymben.2022.10.003

[CR10] Ines M, Dhouha G (2015) Lipopeptide surfactants: production, recovery and pore forming capacity. Peptides 71:100–112 26189973 10.1016/j.peptides.2015.07.006

[CR11] Jiao S, Li X, Yu HM, Yang H, Li X, Shen ZY (2017) In situ enhancement of surfactin biosynthesis in *Bacillus subtilis* using novel artificial inducible promoters. Biotechnol Bioeng 114(4):832–842 27723092 10.1002/bit.26197

[CR12] Lee SY, Lee DY, Kim TY (2005) Systems biotechnology for strain improvement. Trends Biotechnol 23(7):349–358 15923052 10.1016/j.tibtech.2005.05.003

[CR13] Li M, Chen Z, Zhang X, Song Y, Wen Y, Li J (2010) Enhancement of avermectin and ivermectin production by overexpression of the maltose ATP-binding cassette transporter in *Streptomyces avermitilis*. Bioresour Technol 101(23):9228–9235 20655739 10.1016/j.biortech.2010.06.132

[CR14] Li X, Yang H, Zhang D, Li X, Yu H, Shen Z (2015) Overexpression of specific proton motive force-dependent transporters facilitate the export of surfactin in *Bacillus subtilis*. J Ind Microbiol Biotechnol 42(1):93–103 25366377 10.1007/s10295-014-1527-z

[CR15] Mizumoto S, Hirai M, Shoda M (2007) Enhanced iturin A production by *Bacillus subtilis* and its effect on suppression of the plant pathogen *Rhizoctonia solani*. Appl Microbiol Biotechnol 75(6):1267–1274 17453193 10.1007/s00253-007-0973-1

[CR16] Onose S, Ikeda R, Nakagawa K, Kimura T, Yamagishi K, Higuchi O, Miyazawa T (2013) Production of the alpha-glycosidase inhibitor 1-deoxynojirimycin from *Bacillus* species. Food Chem 138(1):516–523 23265519 10.1016/j.foodchem.2012.11.012

[CR17] Qiu Y, Xiao F, Wei X, Wen Z, Chen S (2014) Improvement of lichenysin production in *Bacillus licheniformis* by replacement of native promoter of lichenysin biosynthesis operon and medium optimization. Appl Microbiol Biotechnol 98(21):8895–8903 25085615 10.1007/s00253-014-5978-y

[CR18] Rao Y, Cai D, Wang H, Xu Y, Xiong S, Gao L, Xiong M, Wang Z, Chen S, Ma X (2020) Construction and application of a dual promoter system for efficient protein production and metabolic pathway enhancement in *Bacillus licheniformis*. J Biotechnol 312:1–10 32119878 10.1016/j.jbiotec.2020.02.015

[CR19] Rao Y, Li P, Xie X, Li J, Liao Y, Ma X, Cai D, Chen S (2021) Construction and characterization of a gradient strength promoter library for fine-tuned gene expression in *Bacillus licheniformis*. ACS Synth Biol 10(9):2331–2339 34449215 10.1021/acssynbio.1c00242

[CR20] Tsuge K, Akiyama T, Shoda M (2001) Cloning, sequencing, and characterization of the iturin A operon. J Bacteriol 183(21):6265–6273 11591669 10.1128/JB.183.21.6265-6273.2001 PMC100110

[CR21] Wang J, Qi Q (2009) Synthetic biology for metabolic engineering—a review. Sheng Wu Gong Cheng Xue Bao 25(9):1296–302 19938470

[CR22] Wang M, Yu H, Shen Z (2019) Antisense RNA-based strategy for enhancing surfactin production in *Bacillus subtilis* TS1726 via overexpression of the unconventional biotin carboxylase II to enhance ACCase activity. ACS Synth Biol 8(2):251–256 30702274 10.1021/acssynbio.8b00459

[CR23] Wang F, Zhao J, Li Q, Yang J, Li R, Min J, Yu X, Zheng G, Yu H, Zhai C et al (2020) One-pot biocatalytic route from cycloalkanes to α, ω-dicarboxylic acids by designed* Escherichia col*i consortia. Nat Commun 11(1):5035 33028823 10.1038/s41467-020-18833-7 PMC7542165

[CR24] Wu Q, Zhi Y, Xu Y (2019) Systematically engineering the biosynthesis of a green biosurfactant surfactin by *Bacillus subtilis* 168. Metab Eng 52:87–97 30453038 10.1016/j.ymben.2018.11.004

[CR25] Xia L, Wen J (2022) Available strategies for improving the biosynthesis of surfactin: a review. Crit Rev Biotechnol 43(7):1111–1128 10.1080/07388551.2022.2095252 36001039

[CR26] Xiao J, Peng B, Su Z, Liu A, Hu Y, Nomura CT, Chen S, Wang Q (2020) Facilitating protein expression with portable 5′-UTR secondary structures in *Bacillus licheniformis*. ACS Synth Biol 9(5):1051–1058 32302094 10.1021/acssynbio.9b00355

[CR27] Xu YX, Cai DB, Zhang H, Gao L, Yang Y, Gao JM, Li YY, Yang CL, Ji ZX, Yu J et al (2020) Enhanced production of iturin A in *Bacillus amyloliquefaciens* by genetic engineering and medium optimization. Process Biochem 90:50–57

[CR28] Yaraguppi D, Bagewadi Z, Mahanta N, Singh S, Khan TM, Deshpande SH, Soratur C, Das S, Saikia D (2022) Gene expression and characterization of iturin A lipopeptide biosurfactant from *Bacillus aryabhattai* for enhanced oil recovery. Gels 8(7):403 35877488 10.3390/gels8070403 PMC9319305

[CR29] Yaseen Y, Gancel F, Drider D, Bechet M, Jacques P (2016) Influence of promoters on the production of fengycin in *Bacillus* spp. Res Microbiol 167(4):272–281 26912322 10.1016/j.resmic.2016.01.008

[CR30] Zhan Y, Shi J, Xiao Y, Zhou F, Wang H, Xu H, Li Z, Yang S, Cai D, Chen S (2022) Multilevel metabolic engineering of *Bacillus licheniformis* for *de novo* biosynthesis of 2-phenylethanol. Metab Eng 70:43–54 35038552 10.1016/j.ymben.2022.01.007

[CR31] Zhang X, Lin Y, Wu Q, Wang Y, Chen G (2020) Synthetic biology and genome-editing tools for improving PHA metabolic engineering. Trends Biotechnol 38(7):689–700 31727372 10.1016/j.tibtech.2019.10.006

[CR32] Zhang Z, He P, Cai D, Chen S (2022) Genetic and metabolic engineering for poly-γ-glutamic acid production: current progress, challenges, and prospects. World J Microb Biotechnol 38(11):208 10.1007/s11274-022-03390-6 36030456

[CR33] Zhao H, Shao D, Jiang C, Shi J, Li Q, Huang Q, Rajoka MSR, Yang H, Jin M (2017) Biological activity of lipopeptides from *Bacillus*. Appl Microbiol Biotechnol 101(15):5951–5960 28685194 10.1007/s00253-017-8396-0

[CR34] Zhao M, Huang D, Zhang X, Koffas M, Zhou J, Deng Y (2018) Metabolic engineering of *Escherichia coli* for producing adipic acid through the reverse adipate-degradation pathway. Metab Eng 47:254–262 29625225 10.1016/j.ymben.2018.04.002

[CR35] Zhao S, Xiang B, Yang L, Chen J, Zhu C, Chen Y, Cui J, Hu S, Hu Y (2022) Genetic modifications of critical regulators provide new insights into regulation modes of raw-starch-digesting enzyme expression in *Penicillium*. Biotechnol Biofuels Bioprod 15(1):62 35641999 10.1186/s13068-022-02162-6 PMC9158223

[CR36] Zhu J, Li L, Wu F, Wu Y, Wang Z, Chen X, Li J, Cai D, Chen S (2021) Metabolic engineering of aspartic acid supply modules for enhanced production of bacitracin in *Bacillus licheniformis*. ACS Synth Biol 10(9):2243–2251 34324815 10.1021/acssynbio.1c00154

