# Supplementary material for: Modular metabolic engineering of Bacillus amyloliquefaciens for high-level production of green biosurfactant iturin A
Source: Appl Microbiol Biotechnol. 2024 Apr 27;108:311. doi: 10.1007/s00253-024-13083-9 (PMC11055739; doi:10.1007/s00253-024-13083-9)
Supplement: Supplementary file 1 [file 253_2024_13083_MOESM1_ESM.pdf]

Supplementary Materials

Modular metabolic engineering of *Bacillus amyloliquefaciens* for high level production of green biosurfactant Iturin A

Menglin She [a#], Huijuan Zhou [a#], Wanrong Dong [a], Yuxiang Xu [a], Lin Gao [a, b], Jiaming Gao [c], Yong Yang [a], Zhifan Yang [a], Dongbo Cai [a*], Shouwen Chen [a*]

[a] *State Key Laboratory of Biocatalysis and Enzyme Engineering, Environmental Microbial Technology Center of Hubei Province, College of Life Sciences, Hubei University, Wuhan, 430062, PR China*

[b] *Tobacco Research Institute, Chinese Academy of Agricultural Sciences, Qingdao, 266101, PR China*

[c] *Hubei Corporation of China National Tobacco Corporation, Wuhan, 430000, PR China*

[#] Menglin She and Huijuan Zhou contributed equally to this work.

[*]Corresponding author: Prof. Shouwen Chen and Dr. Dongbo Cai

Tel./fax.: +86 027-88666081.

E-mail address: mel212@126.com (S. Chen) and caidongbo@hubu.edu.cn (D. Cai)

Postal address: 368 Youyi Avenue, Wuchang District, Wuhan 430062, Hubei, PR China

Table S1 The primers used in this research

Primer name	Sequences (5′→3′)
pHY-F	TCTCATTTGCGGCTTCCTTGT
pHY-R	ATTTCATCCCCGCCTTACCTA
T2-L	ACGAATTCCTGCAGCCCGGGGGA
T2-R	TTCTGCGCGTAATCTGCTGCTTGC
P_{udal3}-itu-F1	GGCGAGCTCAACGGCTCATTCGTCTCAT
P_{udal3}-itu-R1	AGTGTCACATCAATATTTCAAATACGCCGCAGAACAT
P_{udal3}-itu-F2	ATGTTCTGCGGCGTATTTGAAATATTGATGTGACACT
P_{udal3}-itu-R2	AAAGGCAAGATTGTTCATTGATCCTTCCTCCTTTAGA
P_{udal3}-itu-F3	TCTAAAGGAGGAAGGATCAATGAACAATCTTGCCTTT
P_{udal3}-itu-R3	GCTCTAGACCGCCATCTTGATGACACG
P_{udal3}-itu-KYF	ATGTAAACGAACAAGCGGGGA
P_{udal3}-itu-KYR	TGATCCTTCCTCCTTTAGA
T2-gabT1-F1	GCTCTAGAGC AATAGCGGTGAGGATAATCGTGC
T2-gabT1-R1	CTCACTTGATTTCCTCCAATAGC
T2-gabT1-F2	GGTATTTGTGGGAACGAAGGAA
T2-gabT1-R2	CGAGCTCG TTGAAAACAAAAACCCCGCCA
T2-gabT1-KYF	CCGCCAAGGCAATAAACAG
T2-gabT1-KYR	TGACGCTTTCTTCCACTCC
P43-F	TGATAGGTGGTATGTTTTCGCTTG
P43-R	CAGTGCGACGGCATTATTGCG
TamyL-F	AAGAGCAGAGAGGACGGATT
TamyL-R	CAGTGCGACGGCATTATTGCG
T2-P43-yngH-F1	GCTCTAGA ATCGTTCGGCCCGGAACATC
T2-P43-yngH-R1	TCCGTCCTCTCTGCTCTTATGTGAAGCACTCCTTTTTCA
T2-P43-yngH-F2	GCTGGACCGTCATCATTAGCGGAATTTCCAATTTCAT
T2-P43-yngH-R2	AATCAGGACTTTTTTAAACATTATATATTCCTCCTTTCTAATATAC
T2-P43-yngH-F3	ATCTAAAGGAGGAAGGATCAATGTTTAAAAAAGTCCTGATT
T2-P43-yngH-R3	GCGAGCTC CTTCTTCTGAATGTACGAT
T2-P_{bacA}-yngH-F1	GCTCTAGA ATCGTTCGGCCCGGAACATC
T2-P_{bacA}-yngH-R1	TCTCGCCGAAATCGCAGGTAATGATGACGGTCCAGC
T2-P_{bacA}-yngH-F2	GCTGGACCGTCATCATTACCTGCGATTTCGGCGAGA
T2-P_{bacA}-yngH-R2	AATCAGGACTTTTTTAAACAATAAAAATTCTCCTTTTT
T2-P_{bacA}-yngH-F3	AAAAAGGAGAATTTTTAT TGTTTAAAAAAGTCCTGATT
T2-P_{bacA}-yngH-R3	GCGAGCTC CTTCTTCTGAATGTACGAT
T2-P_{dual3}-yngH-F1	GCTCTAGA ATCGTTCGGCCCGGAACATC
T2-P_{dual3}-yngH-R1	TAATGATGACGGTCCAGCTTAAGTTAACTTGAGCTCTT
T2-P_{dual3}-yngH-F2	AAGAGCTCAAGTTAACTTAAGCTGGACCGTCATCATTA
T2-P_{dual3}-yngH-R2	AATCAGGACTTTTTTAAACATGATCCTTCCTCCTTTAG
T2-P_{dual3}-yngH-F3	CTAAAGGAGGAAGGATCA TGTTTAAAAAAGTCCTGATT
T2-P_{dual3}-yngH-R3	GCGAGCTC CTTCTTCTGAATGTACGAT
T2- yngH-KYF	CATTGCCTACGGACAGAC

T2- yngH-KYR	TTTGTACTGAACAGTCAC
T2-P_{bacA}-serC-F1	GCTCTAGA TTTCTGTGATAAAGCTCGTA
T2-P_{bacA}-serC-R1	TCTCGCCGAAATCGCAGGTTAAGATGAGGCAAGCCGTTT
T2-P_{bacA}-serC-F2	AAACGGCTTGCCTCATCTTAACCTGCGATTTCGGCGAGA
T2-P_{bacA}-serC-R2	ATTCGTTGTACGTTCCATATAAAAATTCTCCTTTTT
T2-P_{bacA}-serC-F3	AAAAAGGAGAATTTTTAT ATGGAACGTACAACGAAT
T2-P_{bacA}-serC-R3	GCGAGCTCGCTCAGGATGTCGCTTGA
T2-P_{bacA}-serC-KYF	GTCTTAGAACAGTTGGATG
T2-P_{bacA}-serC-KYR	GGACGAGGCTCAGCATAT
T2-P_{bacA}-proA-F1	GGGATCCACTAGTTCTAGACTGGAGCAGTATAAGATTGATG
T2-P_{bacA}-proA-R1	CATGAAATTGGAAATTCCGCTGTAACGCCAGGTATCGC
T2-P_{bacA}-proA-F2	GCGATACCTGGCGTTACAGCGGAATTTCCAATTTCATG
T2-P_{bacA}-proA-R2	CAATTCTTTGTTTTTTCATTATATATTCCTCCTTTCTAA
T2-P_{bacA}-proA-F3	TTAGAAAGGAGGAATATATAATGAAAAAACAAAGAATTG
T2-P_{bacA}-proA-R3	GATCTTTTCTACGAGCTCGTGCCGACCTTTGAGCCT
P_{bacA}-proB-KYF	AATTGCGGTCGTCATCAG
P_{bacA}-proB-KYR	TCATTTGTGTTCCCCCTCG
pgsB-F1	GGGATCCACTAGTTCTAGAGTAACGAGGCTGACGACC
pgsB-R1	CGCTGAACCGTGGATATTTCCGCAATAATGACCTCG
pgsB-F2	CGAGGTCATTATTGCGGAAATATCCACGGTTCAGCG
pgsB-R2	GATCTTTTCTACGAGCTCGTGCTGGCGAATGTAATC
pgsB-KYF	TGGTTACTCATTATAGCCTGTG
pgsB-KYR	AAGGTCATCGGCATTTCT
srfA-F1	CGCGGATCCCAGGCGGTTTGGAGTGTATT
srfA-R1	TTTGCTTCGTCAGGTCGTGCTTGAACCAATCCGTCAGAGGC
srfA-F2	GCCTCTGACGGATTGGTTCAAGCACGA CCTGACGAAGCAAA
srfA-R2	GCTCTAGATCAGCCCGTAACCGAGAACC
srfA-KYF	ATACAATGCCCTGCGAGAA
srfA-KYR	CGGCGGGAACGCAAACAGT
epsA-F1	CGCGGATCCCCGTTTTTCAGTCAGTTTGTTC
epsA -R1	TTTTCTGAATCCCAAATGCCGTAATATACAGCCTCATCCT
epsA -F2	AGGATGAGGCTGTATATTAC GGCATTTGGGATTCAGAAAA
epsA -R2	GCTCTAGACAAAACGACGAGAACGCTTCC
epsA -KYF	TCAGCATATGATTAGTAACGCT
epsA -KYR	ATAAAGGTAAGGAGACATCAC
P_{bacA}-F1	TCTAGAACTAGTGGATCCCCGGTATGACGGCATCTCC
P_{bacA}-R1	TCTCGCCGAAATCGCAGGATGAACGAAAAAAAAGAGA
P_{bacA}-swrC-F2	TCTCTTTTTTTTCGTTCATCCTGCGATTTCGGCGAGA
P_{bacA}-swrC-R2	AGTTAATAATGTGGTTCATATAAAAATTCTCCTTTTT
TamyL-F3	AAAAAGGAGAATTTTTATATGAACCACATTATTAACT
TamyL-R3	GATCTTTTCTACGAGCTCACGGTATCTTCATCAAGTCC
PHY-ycxA-F	GAGAGGAATGTACACATGCGCACGTCTCCCAGG
PHY-ycxA-R	AATCCGTCCTCTCTGCTCTTTTATATTGAATGGTGGGT
PHY-KrsE-F	ATGAAAACTACCGATTCAAAA

PHY-KrsE-R	TCAAAAATTATTTGCTCTTATA
PHY-swrC-F	GAGAGGAATGTACACATGAACCACATTATTAACT
PHY-swrC-R	AAATCCGTCCTCTCTGCTCTTATCGCTTTGCTTAACATC

Table S2 Comparison of iturin A titers produced by HZ-T14 with other iturin A producers

Strain	Titer (g/L)	Strategies	References
B. subtilis ZK0	0.22	Overexpression of *sigA*, *comA*	Zhang et al., 2017
B. amyloliquefaciens LZ-5	0.18	Mutagenesis breeding	Shi et al., 2018
B. subtilis RB14-CS	0.93	Fermentation process optimization	Habe et al., 2019
B. subtilis RB14-CS	4.4	Fermentation process optimization	Mizumoto et al., 2007
B. amyloliquefaciens LL3	0.11	Genetic engineering and Fermentation process optimization	Dang et al., 2019
B. amyloliquefaciens HZ-12	2.01	Genetic engineering and Fermentation process optimization	Xu et al., 2020
B. amyloliquefaciens HZ-12	2.96	Strengthening fatty acid synthesis	Gao et al., 2022
B. amyloliquefaciens HM618	0.11	Fermentation medium optimization and artificial consortia application	Bai et al., 2023
B. amyloliquefaciens HM618	0.07	Co-cultivation with *Corynebacterium glutamicum* to strengthen Pro supply	Chen et al., 2022
B. amyloliquefaciens CX-20	1.95	Pretreatment of fermented rapeseed meal	Wang et al., 2021
B. subtilis ZK-H2	0.85	Fermentation optimization by response surface methodology	Yue et al., 2021
***B. amyloliquefaciens* HZ-T14**	**8.53**	**Rewiring the modules of iturin A synthetase gene cluster, corn starch utilization, precursor and by-product syntheses and iturin A transport**	**This research**

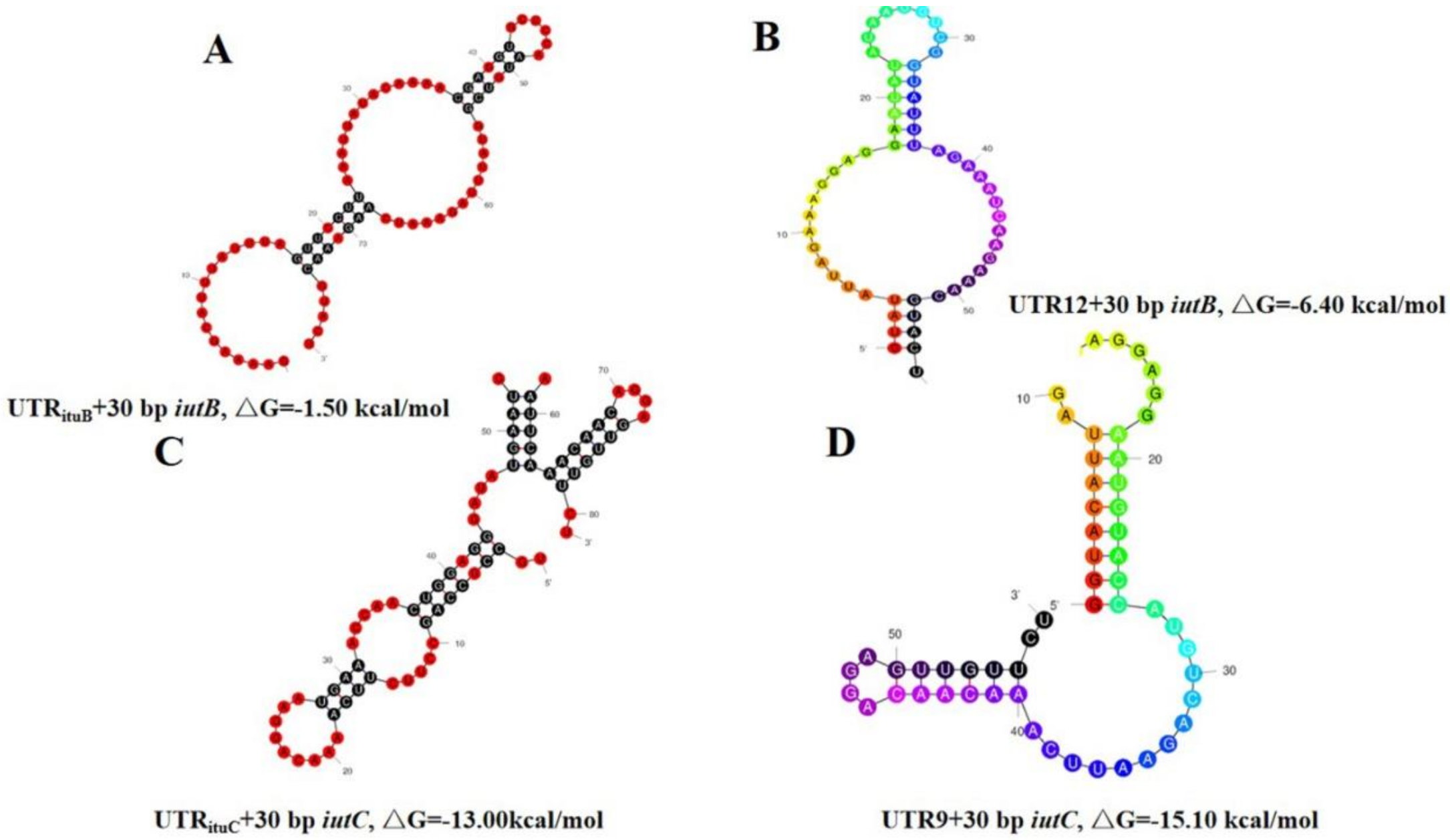

Figure S1 mRNA secondary structure of 5'-UTR with gene *ituB* and *ituC*. **A**: mRNA secondary structure of gene *ituB* with its original 5'-UTR, **B**: The mRNA secondary structure of UTR12 with gene *ituB*, **C**: mRNA secondary structure of gene *ituC* with its original 5'-UTR, **D**: The mRNA secondary structure of UTR12 with gene *ituC*.

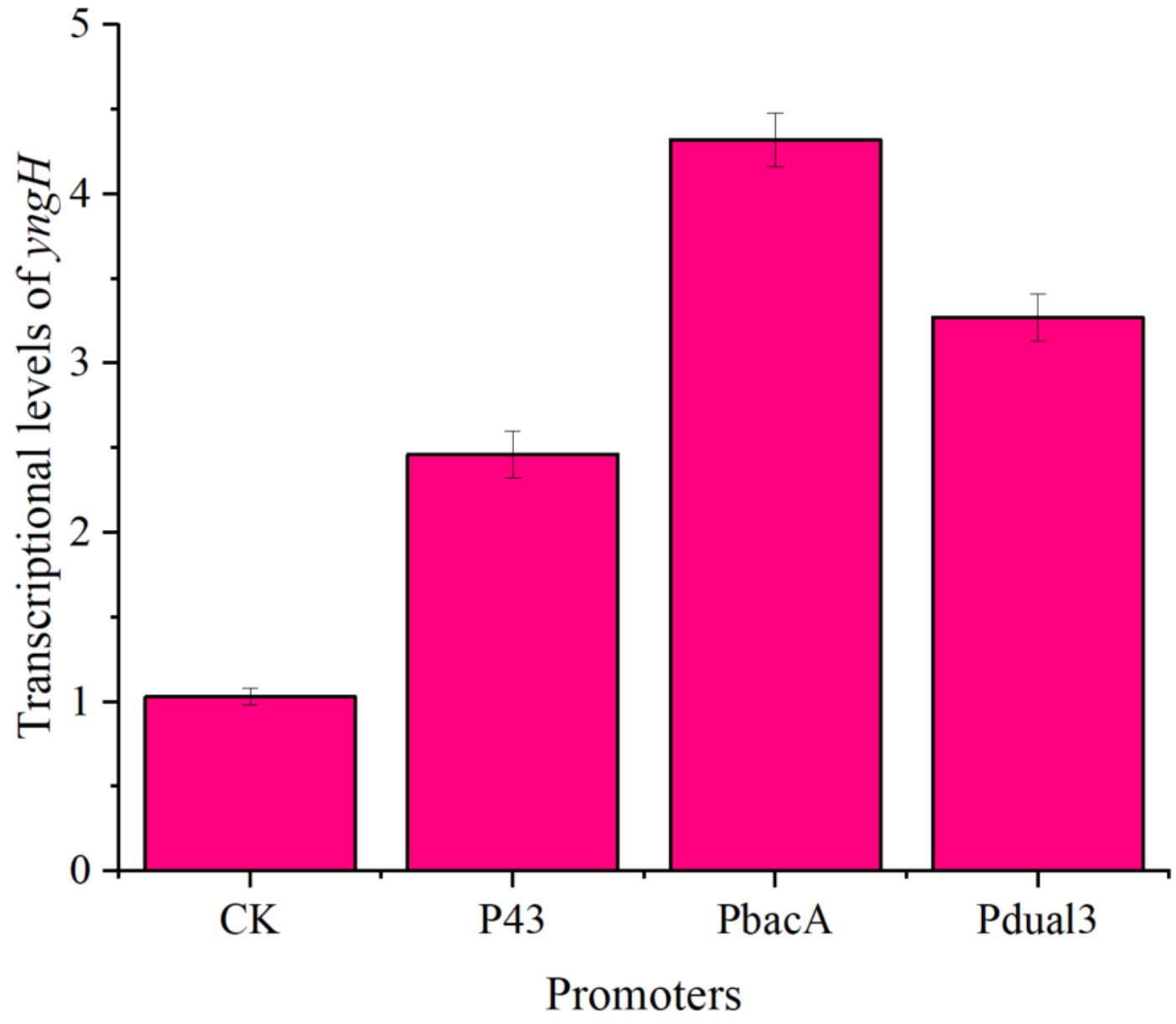

Figure S2 Effects of promoter replacement on the transcriptional levels of *yngH*.
